# Detection and Molecular Characterisation of *Protoparvovirus carnivoran1* in Golden Jackals (*Canis aureus*) in Croatia

**DOI:** 10.3390/v18010123

**Published:** 2026-01-17

**Authors:** Ivona Coric, Gorana Miletic, Dean Konjevic, Ivica Boskovic, Miljenko Bujanic, Alenka Skrinjaric, Snjezana Kovac, Ljubo Barbic, Andreja Jungic, Vladimir Stevanovic

**Affiliations:** 1 Department of Microbiology and Infectious Diseases with Clinic, Faculty of Veterinary Medicine, University of Zagreb, 10000 Zagreb, Croatia; gmiletic@vef.unizg.hr (G.M.);; 2 Department of Veterinary Economics and Epidemiology, Faculty of Veterinary Medicine, University of Zagreb, 10000 Zagreb, Croatia; 3 Department of Animal Production and Biotechnology, Faculty of Agrobiotechnical Sciences, Josip Juraj Strossmayer University of Osijek, 31000 Osijek, Croatia; 4 Hunting and Education Polygon Črnovšćak, Faculty of Veterinary Medicine, University of Zagreb, 10000 Zagreb, Croatia; 5 Department of Virology, Croatian Veterinary Institute, 10000 Zagreb, Croatia

**Keywords:** *Protoparvovirus carnivoran1*, feline panleukopenia virus, phylogenetic analysis, Croatia, golden jackal, VP2 protein

## Abstract

Protoparvoviruses are highly contagious pathogens that cause severe, often fatal diseases in both domestic and wild carnivores. Golden jackal (*Canis aureus*) populations have experienced expansion in recent years, increasingly occupying urban and peri-urban areas. Despite this, they remain largely overlooked in scientific research. This study aimed to detect and characterise *Protoparvovirus carnivoran1* circulating in a golden jackal population in Croatia and to assess their role in the epidemiology of parvovirus infections in companion animals. Small intestines from 55 jackals hunted in 2024 and 2025 were tested for *Protoparvovirus carnivoran1* using real-time PCR. Positive samples were found across all sampling sites, with an overall positivity rate of 40%. Based on characteristic amino acid residues within the VP2 protein, the viruses detected in jackals were classified as feline panleukopenia virus (FPV). Phylogenetic analysis of the VP2 protein demonstrated considerable genetic diversity among strains circulating in Croatia. Additionally, a distinct group was identified, shared exclusively by Croatian domestic cats and golden jackals. Amino acid analysis revealed the novel A91T mutation, found only in jackals, and the E411Q mutation, unique to Croatian FPV strains. Structural modelling of the VP2 protein indicates that the observed mutations are located on the protein surface, within the antibody-binding site. These findings highlight the potential role of wild carnivores in parvovirus epidemiology and underscore the importance of including them in future surveillance and research efforts.

## 1. Introduction

*Protoparvovirus carnivoran1* is a small, non-enveloped, single-stranded DNA virus classified within the family Parvoviridae [1,2]. Its genome is approximately 5.2 kb long and contains two main open reading frames (ORFs). ORF1 encodes the non-structural proteins NS1 and NS2, while ORF2 encodes the structural proteins VP1 and VP2. VP2 plays a key role in determining viral antigenicity and host range. Mutations in VP2 can alter receptor binding and facilitate immune escape. The parvoviral capsid is composed of 60 copies of a single structural protein organised into icosahedral symmetry. The main capsid component, VP2, has an eight-stranded antiparallel β-barrel core, from which approximately two-thirds of the polypeptide chain extends as loop insertions connecting β-strands. These loop regions form spike-like protrusions located at or near the icosahedral threefold axes. The β-barrel core creates a cylindrical depression around the icosahedral fivefold axes [3].

The two most important pathogens in the species *Protoparvovirus carnivoran1* are feline panleukopenia virus (FPV) and canine parvovirus type 2 (CPV-2). FPV was first described in 1928 as a potential causative agent of acute gastroenteritis and leukopenia in cats [4]. FPV infects animals of all ages but particularly young ones, with mortality rates reported between 50% and 80%. Its high pathogenicity has established FPV as one of the most significant viral pathogens in domestic cats [5].

Canine parvovirus is closely related to FPV and is believed to have originated from it [6,7]. The specific ancestral viral strain that gave rise to CPV-2 has not yet been identified. A role for wildlife reservoirs in the emergence of CPV-2 has been proposed, but conclusive evidence remains lacking. Since its emergence, CPV-2 has undergone multiple evolutionary events, resulting in new antigenic variants that have rapidly spread worldwide [7]. In contrast, FPV has remained largely antigenically stable since its discovery, with studies indicating a substantially lower rate of evolution compared to CPV-2 [8].

The *Protoparvovirus carnivoran1* host range is mainly determined by the interaction between VP2 and the transferrin receptor, which facilitates viral entry into host cells [9]. While CPV-2 has a wide host range, FPV has traditionally been considered to be confined to domestic and wild felids and incapable of infecting dogs [10]. However, in 2019, a strain of FPV with the A300P mutation was isolated and demonstrated the ability to infect canines [11].

Changes in host range can significantly influence the epidemiology of infectious diseases, including *Protoparvovirus carnivoran1* infections. For instance, recent studies have documented an expansion of FPV’s host range to include other carnivorous species [12,13,14,15,16]. The significance of changes in host range in the epidemiology of *Protoparvovirus carnivoran1* infections is underscored today, as urbanisation brings wildlife into close contact with humans. Wild animals, like golden jackals, increasingly rely on human-derived food sources [17,18], enabling them to thrive in urban and peri-urban environments [19].

In Croatia, two distinct populations of golden jackal have been identified. The long-established Dalmatian population inhabits coastal areas along the Adriatic Sea and has remained relatively stable. On the other hand, the continental population, once considered extinct, is currently undergoing rapid expansion, forcing jackals to share habitat with humans and domestic animals [20,21]. This may create opportunities for pathogen spillover from the domestic to wild carnivore population, as well as in the opposite direction. Investigating the role of golden jackals in the epidemiology of *Protoparvovirus carnivoran1* is thus essential for understanding parvovirus dynamics in domestic and wild species, as they may serve as bridging hosts.

This study aimed to investigate the circulation of *Protoparvovirus carnivoran1* in golden jackals in continental Croatia and to compare the obtained sequences with strains currently circulating in domestic animals from the same area.

## 2. Materials and Methods

### 2.1. Clinical Samples 

**Figure 1:**
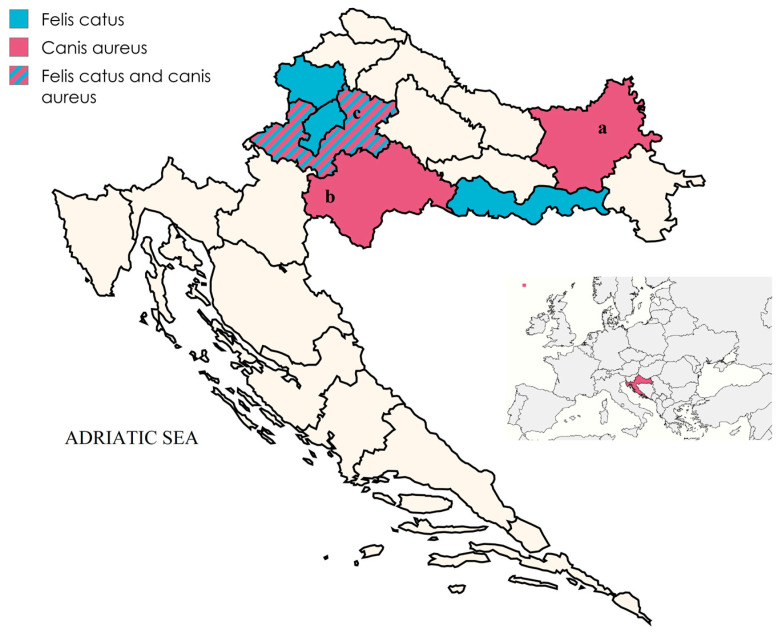
Locations of sampled golden jackals and domestic cats. (a) Osijek-Baranja County, (b) Sisak-Moslavina County, (c) Zagreb County.

As part of a game management plan during 2024 and 2025, 55 small intestine samples were obtained from golden jackals by local hunting associations. These samples were collected from continental Croatia: Osijek-Baranja County, Sisak-Moslavina County and Zagreb County (Figure 1). The samples were submitted to the Faculty of Veterinary Medicine, University of Zagreb, on the same day and stored at −70 °C until subsequent viral DNA extraction. No information on pathological findings, sex or age of animals was available. 

In addition to the jackal samples, 11 archived samples from routine diagnostics at the Virology Laboratory, Faculty of Veterinary Medicine, University of Zagreb, were included in this study. These rectal swabs originated from domestic cats and had previously tested positive for FPV during routine diagnostics. Samples were collected in 2024 and 2025 from continental Croatia (Figure 1). 

### 2.2. DNA Extraction and Polymerase Chain Reaction (PCR) 

Viral DNA from small intestine samples was extracted using the DNeasy Blood and Tissue Kit (QIAGEN, Hilden, Germany) according to the manufacturer’s instructions. Viral DNA from rectal swabs was extracted using the IndiSpin Pathogen Kit (INDICAL BIOSCIENCE, Leipzig, Germany) according to the manufacturer’s instructions.

Viral DNA was detected using a minor groove binder real-time PCR assay, as previously described [22]. Briefly, following the activation of GoTaq^®^ Hot Start Polymerase included in GoTaq^®^ qPCR Master Mix (Promega Corporation, Madison, WI, USA) at 95 °C for 2 min, 45 cycles of a two-step PCR were performed, consisting of denaturation at 95 °C for 30 s and primer annealing/extension at 60 °C for 1 min. Fluorescence was measured during the annealing/extension step, and samples with a cycle threshold value below 35 were considered positive. Reactions were performed on a Rotor-Gene Q real-time cycler (QIAGEN, Hilden, Germany). 

In positive samples, the full-length VP2 gene was amplified using previously published, overlapping PCR primer pairs [23], with PCR conditions optimised for the present study. Following activation of TaKaRa Taq™ DNA Polymerase Hot Start Version (Takara Bio, Shiga, Japan) at 95 °C for 5 min, touchdown PCR was performed with annealing temperatures decreasing from 55 °C to 45 °C over 45 cycles. Primers and the expected amplicon sizes are listed in Supplementary Table S2. PCR products were visualised by electrophoresis on a 1% agarose gel stained with ethidium bromide and viewed under UV illumination using a Uvidoc HD6 (UVITEC, Cambridge, UK). Amplified VP2 segments were subjected to Sanger sequencing using the identical primer pairs (LGC Genomics GmbH, Berlin, Germany). 

### 2.3. Sequence Alignment and Mutation Site Analysis 

Nucleotide sequences obtained in this study were manually visualised and assembled using MEGA version 11.0 [24]. The resulting consensus sequences were compared against the National Centre for Biotechnology Information (NCBI) nucleotide database using the Basic Local Alignment Search Tool 2. 17. 0 (BLAST) [25]. Amino acids at positions 80, 93, 103, 323, 564, and 568 were then analysed to characterise *Protoparvovirus carnivoran1* subtype [26]. 

Amino acid sequences of Croatian FPV isolates were compared to the reference strain CU-4 (GenBank accession number M38246) to identify positions of amino acid substitutions. Additionally, all available VP2 sequences from golden jackals in the NCBI database were retrieved, and mutation sites were compared with those of the Croatian isolates using MEGA software. 

### 2.4. Phylogeny Analysis 

The phylogenetic analysis included full-length VP2 sequences of all 18 Croatian isolates, along with 103 FPV sequences from various countries and time periods retrieved from the NCBI database. A list of all sequences obtained from NCBI is provided in Supplementary Table S3. 

A maximum-likelihood phylogenetic tree of the complete FPV VP2 gene was constructed in IQ-TREE2 with 1000 bootstrap replicates [27]. The Tamura-3 parameter model [28] was selected based on the Akaike Information Criterion and the Bayesian Information Criterion using ModelFinder. The resulting phylogenetic tree was visualised using iTOL software version 7.2.2 [29]. 

### 2.5. Three-Dimensional Modelling Prediction of FPV VP2 Protein Structure 

To assess the position of the amino acid substitution in the three-dimensional structure of the VP2 protein, we modelled these substitutions using the mutagenesis function in PyMOL version 3.1.6 [30]. The crystal structure of FPV (PDB ID: 1FPV) was used for molecular modelling [31]. The modelled structure allowed assessment of structural changes resulting from observed amino acid substitutions.

### 2.6. GenBank Accession Numbers 

All Croatian isolates obtained in this study were submitted to the NCBI database, and accession numbers were generated. A list of all accession numbers is provided in Table 1.

## 3. Results

### 3.1. Detection and Characterisation of Protoparvovirus carnivoran1 

Out of 55 golden jackals, 22 animals (40.0%) tested positive for the presence of *Protoparvovirus carnivoran1* using real-time PCR. This represents the first detection of *Protoparvovirus carnivoran1* in golden jackals in Croatia. Positive cases were detected in all sampled locations. In Zagreb County, 3 out of 9 samples were positive (33.3%), while in Osijek-Baranja County, 11 out of 35 samples tested positive (31.4%). The highest positivity rate was observed in Sisak-Moslavina County, where seven out of 11 samples were positive (63.6%). 

Complete VP2 gene sequences (1755 base pairs, encoding 584 amino acids) were obtained from seven of the positive golden jackals and all domestic cats. Sequences were obtained from golden jackal samples with Ct values ranging from 22.31 to 31.70, whereas samples with higher Ct values did not yield sequences (Supplementary Table S1). Amino acid analysis at positions 80, 93, 103, 323, 564, and 568 [26] indicated that all Croatian sequences examined in this study matched the characteristic amino acid sites of the FPV. Based on NCBI BLAST analysis, it was found that all sequences from the golden jackals and domestic cats exhibited 99–100% similarity to the available FPV sequences.

### 3.2. Phylogenetic Analysis 

**Figure 2:**
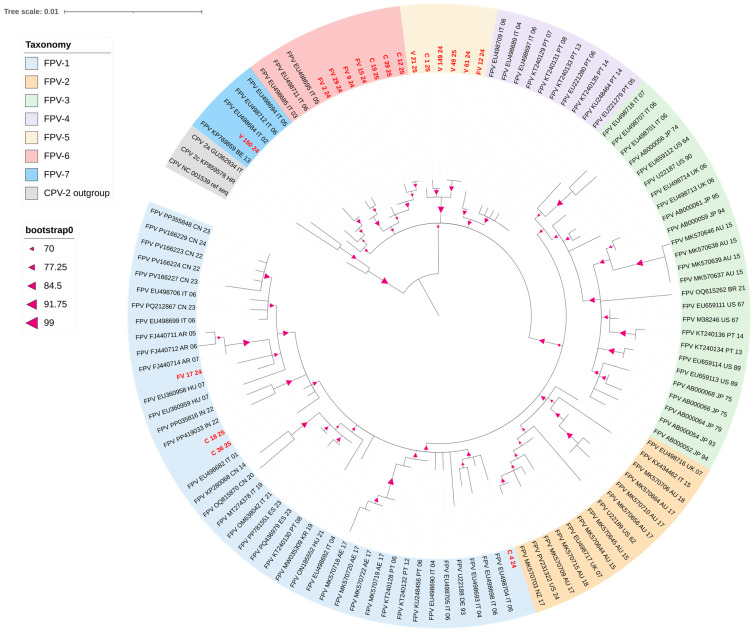
Phylogenetic tree based on the VP2 gene from strains obtained in this study and reference strains. Phylogenetic relationships were calculated using the maximum likelihood method. Bootstrap values, derived from 1000 replicates, appear as magenta triangles; only values of 70 or more are shown. VP2 sequences obtained in this study are written in red. Colours denote group taxonomy. Taxon names consist of an accession number, a two-letter country abbreviation code, and the year of sample collection.

Phylogenetic analysis of the VP2 gene identified seven distinct FPV groups with bootstrap values ranging from 70.00 to 91.75% (Figure 2). CPV-2 strains formed a separate group, with no isolates from this study present in it. Most Croatian isolates were closely related and belonged to the FPV-5, FPV-6, and FPV-7 groups. The FPV-5 group, supported by a bootstrap value over 90, consisted exclusively of Croatian isolates obtained from both domestic cats (V_21_25, V_149_24, V_49_25, V_61_24, FV_12_24) and golden jackals (C_1_25). The FPV-6 group consisted of Croatian domestic cat isolates (FV_2_24, FV_9_24, FV_15_24, FV_24_24) and Croatian golden jackal isolates (C_12_25, C_19_25, C_39_25), which were clustered together with Italian strains obtained from domestic cats. The FPV-7 group contained one Croatian domestic cat isolate (V_150_24), which was most closely related to the domestic cat FPV strains from Italy and Belgium.

In addition to these main clusters, one Croatian domestic cat isolate (FV_17_24) was clustered within the FPV-1 group, along with three Croatian golden jackal isolates (C_4_24, C_18_25, C_36_25).

### 3.3. Amino Acid Mutations in the VP2 Gene 

Croatian golden jackal FPV isolates exhibit several amino acid mutations compared to the FPV CU-4 reference strain. These mutations include A5T, A91T, I101T, and E411Q, as shown in Table 2. Of particular interest, two golden jackal isolates (C_12_25 and C_39_25) carry the A91T mutation, previously reported in FPV strains in golden jackals but not further studied [15,16]. None of the domestic cat isolates carry this mutation; however, one isolate harbours the A91S mutation, which has previously been identified in FPV strains from China [26,32]. The E411Q mutation, also not previously reported, was identified in seven isolates (39%), including both golden jackals (C_12_25, C_19_25, C_39_35) and domestic cats (FV_2_24, FV_9_24, FV_15_24, FV_29_24). The I101T mutation, common in many FPV strains [33], was present in all Croatian isolates, including those from golden jackals.

Comparison of Croatian isolates with partial golden jackal VP2 sequences from NCBI showed that one Italian and one Serbian jackal share the A91T mutation, but none carry the E411Q mutation (Table 2).

### 3.4. Modelling of the VP2 Structure 

**Figure 3:**
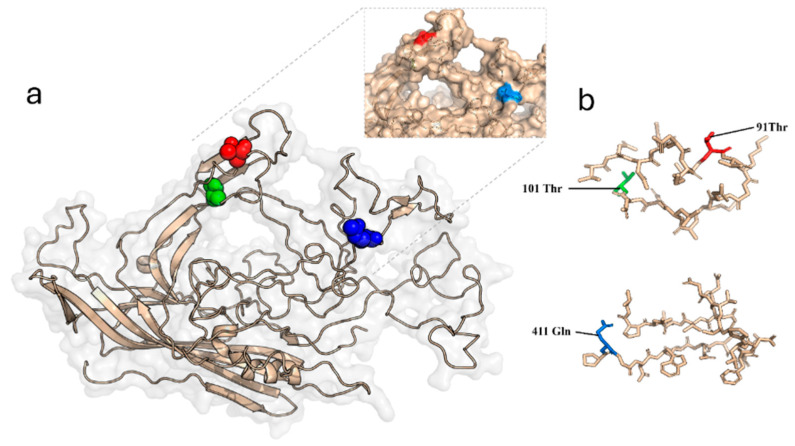
Molecular modelling of capsid protein mutations. (**a**) The cartoon ribbon diagram of the FPV VP2 capsid monomer. The positions of the mutations are marked in blue (411Q), green (101T), and red (91T). Inside the dotted box is a cartoon representation of the FPV capsid protein, highlighting the locations of the mutations. (**b**) Enlarged details from the cartoon ribbon diagram.

Structural VP2 visualisation using PyMOL version 3.1.6.1 revealed the locations of amino acid substitutions at positions 91, 101, and 411 in the three-dimensional structure of VP2 (Figure 3). Residues 91 and 411 are surface-exposed and situated near the threefold spike, in a region referred to as the shoulder [3], whereas residue 101 is buried beneath the surface. Residues 91 and 101 are located within loop one, while residue 411 is positioned within loop four.

## 4. Discussion

In the 20th century, the golden jackal population significantly declined in the Balkan Peninsula due to widespread poisoning campaigns targeting wolves, foxes, and other pests, as well as excessive hunting and habitat loss [34]. By the late 1990s, the population in eastern Croatia began to recover, likely moving in from Bulgaria along the Danube River valley and eventually extending into the Sava and Drava valleys. Jackals primarily inhabit lowland, marshy areas overgrown with shrubs, and the war left many of these areas undisturbed, allowing them to raise offspring successfully. As the population increases, jackals disperse during sexual maturation and during juvenile roaming, spreading into less densely populated areas, often inhabiting the edges of human settlements and living alongside people [20]. Despite this, they are frequently overlooked in the epidemiology of infectious diseases, as evidenced by the lack of information on *Protoparvovirus carnivoran1* infection in jackals.

To this date, the only published reports originated from northeastern Italy in 2022 [16] and from Serbia in 2023 [15]. In both countries and in this study, jackals were infected with FPV, unlike other canid hosts, which are usually infected with CPV-2. FPV cannot efficiently bind to the canine transferrin receptor (TfR), which the virus uses to enter host cells; consequently, FPV cannot infect most canids. A critical factor that facilitated the host barrier jump of the FPV-like virus, leading to the emergence of CPV-2, was its ability to bind canine TfR [9]. Interestingly, jackals seem to retain ancestral features of the transferrin receptor. Their receptors probably represent an intermediate stage between feline-like and canine-like receptors, potentially explaining their susceptibility to FPV [35,36]. 

Our study assessed the presence of *Protoparvovirus carnivoran1* infection in jackal populations along the Danube and Drava (Osijek-Baranja County) and the Sava (Sisak-Moslavina County and Zagreb County) in Croatia. The high overall positivity rate of FPV infection (40%) observed in the Croatian golden jackal population is partially consistent with findings from Italy, which reported a positivity rate of 25%. In contrast, research from Serbia indicated a considerably lower positivity rate of only 4.4%. This discrepancy is somewhat surprising, especially given that genetic testing suggests the Croatian continental jackal population and the Serbian population are, in fact, the same [20]. The authors argued that the low positivity rate in their study may be due to the jackals’ advanced age. Since our research did not include age data for the tested animals, this reasoning cannot be further investigated. A more likely explanation for the observed difference is that they used spleen as the sample material, where viral DNA is detected less frequently than in the intestine [37]. 

The phylogenetic analysis identified seven FPV groups. Croatian golden jackals belonged to the FPV-1 and were closely related to the FPV-5 and FPV-6 groups. All groups included strains from Croatian domestic cats and golden jackals. This suggests that at least some of the FPV-positive jackals may have acquired the virus through direct contact with domestic cats or a shared source of infection, such as a contaminated environment or other susceptible hosts. This is further supported by amino acid analysis of isolates in this study, which revealed that Croatian domestic cats and golden jackals share the previously unreported E411Q and I101T mutations. The E411Q mutation appears to be unique to Croatian strains, potentially indicating an independent evolutionary event among the local population. This assumption is further supported by the presence of the FPV-5 group, which includes only Croatian isolates. Three Croatian jackals belonged to the phylogenetically distant FPV-1 group, along with one Croatian domestic cat. The cat exhibited an A91S mutation previously described as the predominant strain in China [26,32]. Since this mutation has not been reported outside China, it appears unlikely that this case was imported from there. There is a possibility that FPV strains share a common mutation pattern, leading to sequences from various continents converging. Regardless, our research indicates that the viral population currently circulating in Croatia is not uniform and that multiple introductions of the virus may have occurred. A high diversity of FPV strains in jackals has previously been reported in both Italy and Serbia. Research from Italy attributed this diversity either to cross-species transmission or to different origins of jackals repopulating the area. Although Croatia’s continental region was repopulated in the 1990s after jackals were brought to the brink of extinction, the population shows low genetic diversity [38]. As a result, the latter explanation is less likely for FPV diversity in jackals. 

The data from this study support the idea of cross-species transmission facilitated by host adaptation. In line with this, the previously undescribed A91T substitution, found exclusively in jackals, may reflect an evolutionary process unique to this host, as the structural regions surrounding residues 93, 300, and 323 are known to contribute to host range determination [39,40,41,42,43]. These residues are part of surface-exposed loop regions that influence differences in antigenicity, host range, and tissue tropism. The A91T mutation in Loop 1, which, with Loops 2 and 4, forms the spike of the threefold capsid axis and contributes to the formation of the antigen A site, one of two major antigenic sites of FPV [41,44].

The E411Q mutation, which had not previously been reported, was identified in both golden jackals and domestic cats. E411Q is located in Loop 4. As antigen A serves both as the site for host receptor binding and as a target for virus neutralisation, mutations at this position are likely to promote host adaptation and help the virus evade the immune response. However, experimental studies are required to determine the biological significance of these changes. 

Although the limited number of analysed sequences precludes more definitive conclusions, our results demonstrate considerable genetic diversity among Croatian FPV strains. The high FPV positivity rate, together with the unique features of their transferrin receptor, indicates that golden jackals are highly susceptible to FPV and may serve both as a reservoir and a bridging host, facilitating virus maintenance and transmission between wildlife and domestic animals. 

## 5. Conclusions

Our study, for the first time, confirmed the presence of FPV in the Croatian golden jackal population, with a high positivity rate among the tested animals. The data reveal a certain limited degree of heterogeneity among the FPV strains circulating in Croatia, including a distinctive Croatian strain. The detection of the A91T amino acid mutation unique to jackals, along with mutations shared by both domestic cats and jackals, highlights the importance of wildlife research for the understanding of the epidemiology of parvoviruses.

## 6. Research Limitations 

The main limitation of the study was the small number of golden jackal sequences, which restricted the ability to draw more definitive conclusions about the significance of the observed data. Data on sex, age and pathological findings would allow further understanding of *Protoparvovirus carnivoran1* infection epidemiology in golden jackals and its clinical relevance. Additionally, obtaining complete viral genomes would allow a more detailed characterisation of the detected viruses. Finally, further experimental studies are needed to fully understand the importance of the mutations observed in this research.

## Figures and Tables

**Table 1:** Host, location, and collection year of Croatian isolates used in the study.

Accession Number	Isolate	Host	County	Year
PX610152	C_4_24	*Canis aureus*	Zagreb	2024
PX610153	C_1_25	*Canis aureus*	Zagreb	2025
PX610154	C_12_25	*Canis aureus*	Osijek-Baranja	2025
PX610155	C_18_25	*Canis aureus*	Osijek-Baranja	2025
PX610156	C_19_25	*Canis aureus*	Osijek-Baranja	2025
PX610157	C_36_25	*Canis aureus*	Osijek-Baranja	2025
PX610158	C_39_25	*Canis aureus*	Sisak-Moslavina	2025
PX610159	FV_2_24	*Felis catus*	Krapina-Zagorje	2024
PX610162	FV_9_24	*Felis catus*	Zagreb	2024
PX610161	FV_12_24	*Felis catus*	Zagreb City	2024
PX610160	FV_15_24	*Felis catus*	Zagreb	2024
PX610167	FV_17_24	*Felis catus*	Brod-Posavina	2024
PX610168	FV_29_24	*Felis catus*	Zagreb City	2024
PX610165	V_21_25	*Felis catus*	Zagreb City	2025
PX610163	V_49_25	*Felis catus*	Zagreb City	2025
PX610164	V_61_24	*Felis catus*	Zagreb City	2024
PX610166	V_149_24	*Felis catus*	Zagreb City	2024
PX610169	V_150_24	*Felis catus*	Zagreb	2024

**Table 2:** Comparison of amino acid residues in the VP2 gene of FPV isolates from this study and partial golden jackal isolates from NCBI. N/A, not applicable.

Accession Number	Isolate	Country	Host	Isolation Source	Amino Acid and Position in VP2			
					5	91	101	411
PV815770	FPV_FVM_38	Serbia	Canis aureus	spleen	N/A	Thr	Thr	Glu
OR236706	SRB-Jackal 101/2023	Serbia	Canis aureus	spleen	N/A	N/A	N/A	Glu
OR236705	SRB-Jackal 56/2022	Serbia	Canis aureus	spleen	N/A	N/A	N/A	Glu
OR236704	SRB-Jackal 49/2022	Serbia	Canis aureus	spleen	N/A	N/A	N/A	Glu
OP587998	IZSVe_21/48559-5_golden_jackal_ITA	Italy	Canis aureus	intestine	Ala	Ala	Thr	Glu
OP588006	IZSVe_21/29010-6_golden_jackal_ITA	Italy	Canis aureus	intestine	Ser	Thr	Thr	Glu
OP595745	IZSVe_22/6391-6_golden_jackal_ITA	Italy	Canis aureus	intestine	Ala	Ala	Thr	Glu
PX610152	C_4_24	Croatia	Canis aureus	intestine	Ala	Ala	Thr	Glu
PX610153	C_1_25	Croatia	Canis aureus	intestine	Thr	Ala	Thr	Glu
PX610154	C_12_25	Croatia	Canis aureus	intestine	Ala	Thr	Thr	Gln
PX610155	C_18_25	Croatia	Canis aureus	intestine	Ala	Ala	Thr	Glu
PX610156	C_19_25	Croatia	Canis aureus	intestine	Ala	Ala	Thr	Gln
PX610157	C_36_25	Croatia	Canis aureus	intestine	Ala	Ala	Thr	Glu
PX610158	C_39_25	Croatia	Canis aureus	intestine	Ala	Thr	Thr	Gln
PX610159	FV_2_24	Croatia	Felis catus	rectal swab	Ala	Ala	Thr	Gln
PX610162	FV_9_24	Croatia	Felis catus	rectal swab	Ala	Ala	Thr	Gln
PX610161	FV_12_24	Croatia	Felis catus	rectal swab	Ala	Ala	Thr	Glu
PX610160	FV_15_24	Croatia	Felis catus	rectal swab	Ala	Ala	Thr	Gln
PX610167	FV_17_24	Croatia	Felis catus	rectal swab	Ala	Ser	Thr	Glu
PX610168	FV_29_24	Croatia	Felis catus	rectal swab	Ala	Ala	Thr	Gln
PX610165	V_21_25	Croatia	Felis catus	rectal swab	Ala	Ala	Thr	Glu
PX610163	V_49_25	Croatia	Felis catus	rectal swab	Ala	Ala	Thr	Glu
PX610164	V_61_24	Croatia	Felis catus	rectal swab	Ala	Ala	Thr	Glu
PX610166	V_149_24	Croatia	Felis catus	rectal swab	Ala	Ala	Thr	Glu
PX610169	V_150_24	Croatia	Felis catus	rectal swab	Ala	Ala	Thr	Glu

## Data Availability

The authors declare that the data supporting the findings of this study are available within the article. Additional information is available from the authors upon reasonable request.

## Supplementary Materials

The following supporting information can be downloaded at: https://www.mdpi.com/article/10.3390/v18010123/s1, Table S1: Sampled golden jackals Ct values. Table S2: Sequence and position of the oligonucleotides used in the study. Table S3: Feline panleukopenia virus sequences from GenBank included in the phylogenetic analysis.

## Abbreviations

The following abbreviations are used in this manuscript:

FPV	Feline panleukopenia virus
CPV-2	Canine parvovirus type 2
TfR	Transferrin receptor
BLAST	Basic Local Alignment Search Tool
NCBI	National Centre for Biotechnology Information
ORF	Open reading frame
PCR	Polymerase Chain Reaction
