# Supplementary material for: Detection and Molecular Characterisation of Protoparvovirus carnivoran1 in Golden Jackals (Canis aureus) in Croatia
Source: Viruses. 2026 Jan 17;18(1):123. doi: 10.3390/v18010123 (PMC12846582; doi:10.3390/v18010123)
Supplement: Supplementary file 1 [file viruses-18-00123-s001.zip › viruses-4098139-supplementary.pdf]

Table S1. Sampled golden jackals Ct values

No.	Isolate	Ct value
1	C-1-24	negative
2	C-2-24	negative
3	C-3-24	negative
4	C-4-24	28,22
5	C-5-24	negative
6	C-6-24	32,51
7	C-7-24	32,16
8	C-8-24	negative
9	C-9-24	34,30
10	C-1-25	29,89
11	C-2-25	34,43
12	C-3-25	negative
13	C-4-25	negative
14	C-5-25	negative
15	C-6-25	32,33
16	C-7-25	negative
17	C-8-25	negative
18	C-9-25	negative
19	C-10-25	negative
20	C-11-25	31,90
21	C-12-25	25,36
22	C-13-25	35,90
23	C-14-25	34,49
24	C-15-25	negative
25	C-16-25	35,14
26	C-17-25	34,93
27	C-18-25	29,19
28	C-19-25	29,64
29	C-20-25	negative
30	C-21-25	negative
31	C-22-25	36,04
32	C-23-25	31,80
33	C-24-25	32,15
34	C-25-25	33,57
35	C-26-25	36,04
36	C-27-25	negative
37	C-28-25	33,70
38	C-29-25	negative
39	C-30-25	negative
40	C-31-25	negative
41	C-32-25	36,02
42	C-33-25	36,29
43	C-34-25	negative
44	C-35-25	negative
45	C-36-25	28,98
46	C-37-25	32,10
47	C-38-25	32,10
48	C-39-25	22,70

49	C-40-25	negative
50	C-41-25	negative
51	C-42-25	negative
52	C-43-25	35,55
53	C-44-25	37,08
54	C-45-25	negative
55	C-46-25	32,85

Table S2. Sequence and position of the oligonucleotides used in the study

Name	Sequence (5'-3')	Amplicon size (bp)	Purpose	Refrence
V51-F	CCAACTAAAAGAAGTAAACC	756	Genome sequencing	Horiuchi et al., 1998
V52-R	ATTAATGTTCTATCCCATTG		Genome sequencing	
V5-F	AGCTATGAGATCTGAGACA	1088	Genome sequencing	
V56-R	AATTGGATTCCAAGTATGAG		Genome sequencing	
V22-F	TGTCAAAATAATTGTCCTG	467	Genome sequencing	
V41-R	ATTGTATACCATATAACAAACC		Genome sequencing	
FPV/CPV-For	ACAAGATAAAAGACGTGGTGTAACTCAA	83	Detection	Decaro et al., 2008
FPV/CPV-Rev	CAACCTCAGCTGGTCTCATAATAGT		Detection	
FPV-Pb	VIC – ATGGGAAATACAGACTATAT – MGB		Detection	
CPV-Pb	FAM – ATGGGAAATACAAACTATAT – MGB		Detection	

Table S3. Feline panleukopenia virus sequences from GenBank included in the pylogenetic analysis

Number	Isolate/Strain	Country of Orgin	Accession Number	Host	Year
1	AO1	Japan	AB000052	Felis catus	1994
2	Fukagawa	Japan	AB000054	Felis catus	1993
3	Obihiro	Japan	AB000056	Felis catus	1974
4	Som1	Japan	AB000059	Felis catus	1994
5	Som4	Japan	AB000061	Felis catus	1995
6	TU12	Japan	AB000064	Felis catus	1979
7	TU2	Japan	AB000066	Felis catus	1975

8	TU4	Japan	AB000068	Felis catus	1975
9	FPLV/cat/46912/PT05	Portugal	EU221279	Felis catus	2005
10	FPLV/cat/39897/PT06	Portugal	EU221280	Felis catus	2006
11	PT020/06	Portugal	KT240128	Felis catus	2006
12	PT001/07	Portugal	KT240129	Felis catus	2007
13	PT005/08	Portugal	KT240130	Felis catus	2008
14	PT022/08	Portugal	KT240131	Felis catus	2008
15	PT183/12	Portugal	KT240132	Felis catus	2012
16	PT083/13	Portugal	KT240133	Felis catus	2013
17	PT210/13	Portugal	KT240134	Felis catus	2013
18	PT264/14	Portugal	KT240135	Felis catus	2014
19	PT271/14	Portugal	KT240136	Felis catus	2014
20	PT001/06	Portugal	KU248456	Felis catus	2006
21	PT265/14	Portugal	KU248464	Felis catus	2014
22	933/07	Hungary	EU360958	Felis catus	2007
23	1335/07	Hungary	EU360959	Felis catus	2007
24	FPV_1724_HU	Hungary	ON185552	Felis catus	2021
25	198/01	Italy	EU498682	Felis catus	2001
26	103/02	Italy	EU498684	Felis catus	2002
27	150/03	Italy	EU498685	Felis catus	2003
28	300/03	Italy	EU498687	Felis catus	2003
29	134/04-1	Italy	EU498688	Felis catus	2004
30	134/04-2	Italy	EU498689	Felis catus	2004
31	134/04-3	Italy	EU498690	Felis catus	2004
32	134/04-5	Italy	EU498691	Felis catus	2004
33	143/04	Italy	EU498692	Felis catus	2004
34	355/04	Italy	EU498693	Felis catus	2004
35	20/05.	Italy	EU498694	Felis catus	2005
36	119/05	Italy	EU498695	Felis catus	2005
37	42/06-G1	Italy	EU498697	Felis catus	2006
38	42/06-G2	Italy	EU498698	Felis catus	2006
39	42/06-G3	Italy	EU498699	Felis catus	2006
40	42/06-G4	Italy	EU498700	Felis catus	2006
41	42/06-G5	Italy	EU498701	Felis catus	2006
42	42/06-G6	Italy	EU498702	Felis catus	2006
43	42/06-G8	Italy	EU498704	Felis catus	2006

44	42/06-G10	Italy	EU498705	Felis catus	2006
45	42/06-G11	Italy	EU498706	Felis catus	2006
46	42/06-12	Italy	EU498707	Felis catus	2006
47	42/06-G16	Italy	EU498709	Felis catus	2006
48	42/06-18	Italy	EU498711	Felis catus	2006
49	42/06-G19	Italy	EU498712	Felis catus	2006
50	443/07	Italy	EU498718	Felis catus	2007
51	FPV_IZSSI_42807_15	Italy	KX434462	Felis catus	2015
52	245-1478_FPLV_BADGER_2019_ITALY	Italy	MT274378	Meles meles	2019
53	ITA/2023/bear/74	Italy	OR602717	Ursus arctos marsicanus	2023
54	ITA/2021/164-1	Italy	OM638042	Canis lupus familiaris	2021
55	97/06-10	United Kingdom	EU498713	Felis catus	2006
56	97/06-11	United Kingdom	EU498714	Felis catus	2006
57	50/07-1	United Kingdom	EU498716	Felis catus	2007
58	50/07-2	United Kingdom	EU498717	Felis catus	2007
59	FVP-3.us_67	USA	EU659111	Felis catus	1967
60	CU-4	USA	M38246	NA	1967
61	D2410482	USA	PV231322	Procyon lotor	2024
62	FPV-23	USA	U22187	Felis sylvestris	1990
63	FPV-4.us_64	USA	EU659112	Felis catus	1964
64	FPV-d	USA	U22189	Felis sylvestris	1964
65	FPV-8a.us_89	USA	EU659113	Puma concolor	1989
66	FPV-8b.us_89	USA	EU659114	Puma concolor	1089
67	FPV-kai.us.06	USA	EU659115	Felis catus	2006
68	FPV_ARG05	Argentina	FJ440711	Felis catus	2005
69	FPV_ARG06	Argentina	FJ440712	Felis catus	2006
70	FPV_ARG08	Argentina	FJ440714	Felis catus	2007
71	MG132167A	Belgium	KP769859	Felis catus	2013
72	FPV_C7_AUS_Mildura_09/2015	Australia	MK570637	Felis catus	2015
73	FPV_C5_AUS_Mildura_08/2015	Australia	MK570638	Felis catus	2015
74	FPV_C4_AUS_Mildura_07/2015	Australia	MK570639	Felis catus	2015
75	FPV_CPS_AUS_Sydney_11/2015	Australia	MK570644	Felis catus	2015

76	FPV_CPS_AUS_Concord_11/2015	Australia	MK570645	Felis catus	2015
77	FPV_31_AUS_Fern_Tree_Gully_Vic_11/2015	Australia	MK570646	Felis catus	2015
78	FPV_89_R_AUS_Syd_01/2017	Australia	MK570656	Felis catus	2017
79	FPV_109_A_AUS_Syd_01/2017	Australia	MK570664	Felis catus	2017
80	FPV_251_AUS_Melb_Vic_2/2018	Australia	MK570706	Felis catus	2018
81	FPV_254_AUS_AcaciaG_Syd_12/2017	Australia	MK570709	Felis catus	2017
82	FPV_255_AUS_AcaciaG_Syd_12/2017	Australia	MK570710	Felis catus	2017
83	FPV_260_R_AUS_Melb_Vic_02/18	Australia	MK570715	Felis catus	2018
84	FPV_223_NZ_06/2017	New Zeland	MK570703	Felis catus	2017
85	Dubai/United Arab Emirates/Felis catus/2017	UAE	MK570718	Felis catus	2017
86	Dubai/United Arab Emirates/Felis catus/2017	UAE	MK570719	Felis catus	2017
87	Dubai/United Arab Emirates/Felis catus/2017	UAE	MK570720	Felis catus	2017
88	Dubai/United Arab Emirates/Felis catus/2017	UAE	MK570722	Felis catus	2017
89	19SP_CK-8	South Korea	MW035309	Canis lupus familiaris	2019
90	UFU/USP16	Brazil	OQ615262	Felis catus	2021
91	FPV/INDIA/MZ33	India	PP035816	Felis catus	2022
92	FPV/INDIA/MZ27	India	PP419033	Felis catus	2022
93	XJ20220223	China	PV166223	Felis catus	2022
94	XJ20220328	China	PV166224	Felis catus	2022
95	XJ20230605	China	PV166227	Felis catus	2023
96	XJ20240205	China	PV166229	Felis catus	2024
96	FPV-6	China	OQ815870	Felis catus	2020
98	HRB-CS1	China	KP280068	Felis catus	2014
99	GSW1-SH2023	China	PP355848	Nyctereutes procyonoides	2023
100	XJ-URC-6	China	PQ212867	Felis catus	2023
101	LG100_Iberian lynx_Spain	Spain	PP781551	Iberian lynx	2023
102	LG15_cat_Spain	Spain	PQ436979	Felis catus	2023
103	FPV-377	Germany	U22188	Felis sylvestris	1993
104	CPV-N	NA	NC_001539	Canis lupus familiaris	NA
105	12/08-B	Italy	GU362934	Canis lupus familiaris	2008

106	HR859	Croatia	KP859578	Canis lupus familiaris	2014
